# Adénome à prolactine induit par les antipsychotiques

**DOI:** 10.11604/pamj.2015.22.341.8235

**Published:** 2015-12-10

**Authors:** Ilyes Marrag, Kilani Hajji

**Affiliations:** 1 Service de Psychiatrie, CHU Taher Sfar, Mahdia, 5100 Mahdia, Tunisie

**Keywords:** Hyperprolactinémie, antipsychotiques, adénome, hyperprolactinemia, antipsychotics, adenoma

## Image en médecine

L'hyperprolactinémie, un trouble endocrine fréquent mais largement sous-estimé, peut être due à diverses causes parmi lesquelles figure le traitement par de nombreux médicaments. Les antipsychotiques jouent en particulier un rôle important dans leur survenue. Leur potentiel hyperprolactinémiant est néanmoins variable, mettant en jeu des mécanismes complexes. La fréquence des signes cliniques est le plus souvent corrélée à l’élévation de la prolactinémie mais l'hyperprolactinémie est parfois asymptomatique. Les manifestations cliniques relèvent principalement de troubles sexuels, de troubles du cycle menstruel et de galactorrhée, en plus d'effets à long terme. Ces signes ne sont pas toujours évoqués par les patients ce qui aboutit à une sous-estimation de la prévalence des hyperprolactinémie. Le risque de développer un adénome à prolactine parait étroitement lié à une élévation significative de la prolactinémie au delà de 150 ng/ml. Nous rapportons l'observation de deux patientes qui ont été inclues dans une étude transversale sur 6 mois, portant sur tous les patients suivis à la consultation et traités par un seul antipsychotique depuis 12 semaines à posologie stable. Un dosage de la prolactinémie plasmatique a été réalisé et confirmé par un deuxième dosage en cas d'anomalie objectivée au premier bilan. Une Imagerie par Résonance Magnétique (IRM) hypophysaire a été demandée pour les patients présentant une prolactinémie supérieure à 150 ng/ml. Ces derniers ont bénéficié d'une IRM hypophysaire révélant 2 cas de macroadénomes. Les deux patientes ont bénéficié d'un avis spécialisé des endocrinologues qui ont cosigné la prescription de la Bromocriptine (Parlodel^®^) avec augmentation progressive de la posologie et monitorage du taux plasmatique de la prolactine dans trois mois ainsi qu'une surveillance des effets psychiatrique vue le risque de décompensation sous dopathérapie. La prescription des agonistes dopaminergiques dans le traitement des hyperprolactinémie doit être prudente dans des cas exceptionnels vue la possibilité de l'aggravation trouble mental sous jacent.

**Figure 1:**
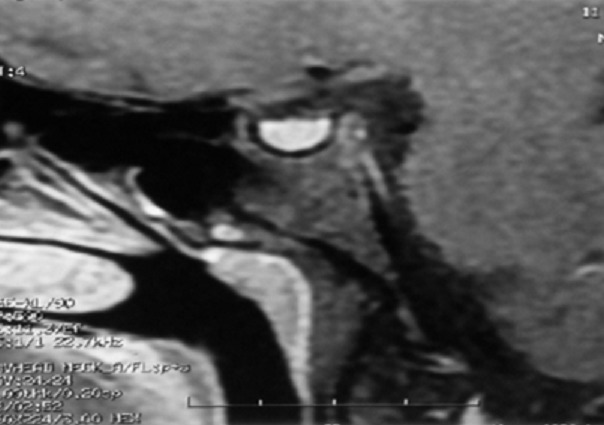
IRM hypophysaire: coupe sagittale en séquence T1 montrant un adénome hypophysaire

